# Built environment microbiomes transition from outdoor to human-associated communities after construction and commissioning

**DOI:** 10.1038/s41598-023-42427-0

**Published:** 2023-09-22

**Authors:** Gregory R. Young, Angela Sherry, Darren L. Smith

**Affiliations:** 1 Department of Applied Sciences, Faculty of Health and Life Sciences, Northumbria University, Newcastle, NE1 8ST UK; 2 Hub for Biotechnology in the Built Environment, Northumbria University, Newcastle, NE1 8ST UK

**Keywords:** Microbiome, Microbial ecology, Ecology, Microbiology

## Abstract

The microbiota of the built environment is linked to usage, materials and, perhaps most importantly, human health. Many studies have attempted to identify ways of modulating microbial communities within built environments to promote health. None have explored how these complex communities assemble initially, following construction of new built environments. This study used high-throughput targeted sequencing approaches to explore bacterial community acquisition and development throughout the construction of a new build. Microbial sampling spanned from site identification, through the construction process to commissioning and use. Following commissioning of the building, bacterial richness and diversity were significantly reduced (P < 0.001) and community structure was altered (R_2_ = 0.14; P = 0.001). Greater longitudinal community stability was observed in outdoor environments than indoor environments. Community flux in indoor environments was associated with human interventions driving environmental selection, which increased 10.4% in indoor environments following commissioning. Increased environmental selection coincided with a 12% reduction in outdoor community influence on indoor microbiomes (P = 2.00 × 10^–15^). Indoor communities became significantly enriched with human associated genera including *Escherichia*, *Pseudomonas*, and *Klebsiella* spp. These data represent the first to characterize the initial assembly of bacterial communities in built environments and will inform future studies aiming to modulate built environment microbiota.

## Introduction

Humans spend roughly 87% of their time in indoor environments^1^. Substantial proportions of the remaining 13% of time is spent in other built environment (e.g. commuting in towns/cities). Recent studies have explored the composition of microbial communities in these built environments, specifically focusing on indoor air^2,3^ as well as common household and public surfaces^4–7^. These studies have shown distinct microbial communities across different built environments, even within individual buildings. Microbial colonization of these environments is related to space utilization^8^, materials used^9^ and ventilation strategies^10^.

As a product of these new indoor environments, the multitudes of microbes living around and inside of humans differ from those of our ancestors or those living in more rural environments^11,12^, which can impact on health. As such, the microbiome of the built environment has been widely studied in relation to long-term health^13–15^ and nosocomial infections^16–19^. In the same way our microbial neighbors can influence human health, human (or non-human) occupancy may also influence the microbial communities of the built environment^10,20^.

The concept of the microbiome as a malleable entity, which can be altered to reduce likelihood of disease onset, is not new^8,21^. However, to make informed interventions on built environment microbiotas to promote human health we must first develop an understanding of how these complex networks react to environmental change. Like any living system, microbial environments favour homeostasis^22^. Similarly to humans, the microbial communities of buildings develop over time. Unlike humans, buildings are not created in a sterile environment^23^. While others have explored the development of microbiota in hospital environments following public access^24^, no studies to date have explored the acquisition and longitudinal development of the built environment microbiota across the entire construction and commissioning process of a new building. Instead, studies have often focused on cross-sectional analyses between one built environment and another, identifying greater diversity in infrequently cleaned homes or homes where canines lived alongside humans^5^. While purely cross-sectional studies are well suited to discount the influence of environmental variables such as occupancy at sampling or cleaning regularity, they fail to capture the influence of seasonality on the microbial communities explored^25^. Community temporality is especially important when exploring the longitudinal development of built environment microbiota.

*The OME* is an experimental building in Newcastle upon Tyne, England. It was built as part of a Research England Expanding Excellence in England, funded Hub for Biotechnology in the Built Environment^26^, and facilitates microbial experimentation and architectural exhibition. In this study we employed longitudinal sampling to define timeframes of microbial acquisition in the built environment by conducting a temporal study at high sampling depth within a single, purpose-built building, *The OME*. We primarily aimed to quantify the time taken for stable microbial communities to engraft in newly erected structures and assess how commissioning the building influenced the microbial communities as specialist locations (e.g. kitchens & hallways) were used for their specific purpose.

## Results

**Figure 1:**
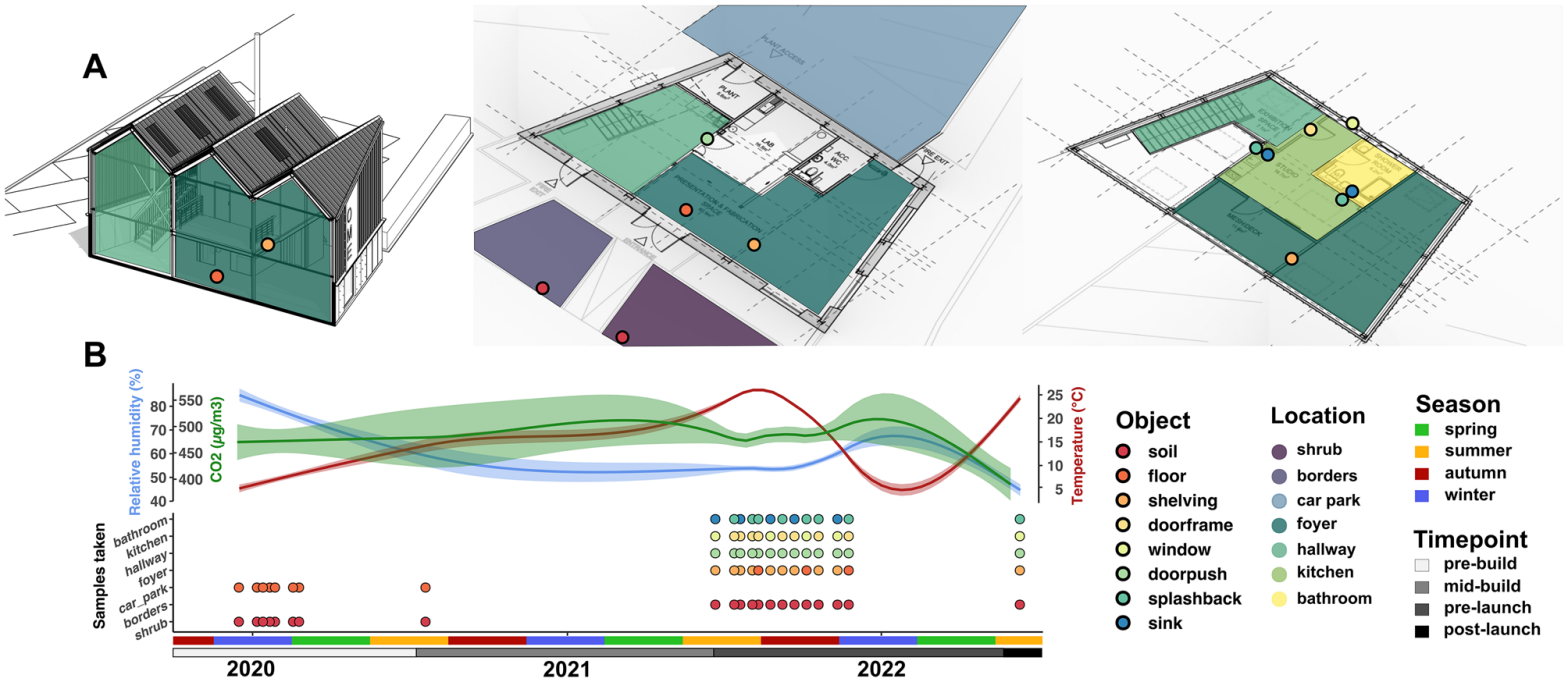
illustrates the locations (**A**) and longitudinal nature (**B**) of sampling employed in this study. A 3D render of the OME building as well as ground and first floor floorplans illustrate sampling locations interrogated during this study (**A**). Longitudinal environmental data including relative humidity (blue), CO_2_ levels (green) and temperature (red) records collected as well as sample types taken at each sampling timepoint are also illustrated (**B**). Shaded areas around humidity, CO_2_ and temperature trendlines represent the 95% confidence interval associated with each environmental record (top panel). Each point on the lower panel represents an individual sampling timepoint, coloured by object and arranged by location on the y axis. The impact of Covid-19 on sampling continuation is apparent in the scarcity between spring 2020 and summer 2021. Humidity, temperature and CO_2_ records were only taken during sampling timepoints and were therefore not recorded during this same period.

Over a study period of 36 months, 439 microbial community samples were collected from The OME site (Supplementary Table 1). Samples were taken from 8 different objects (soil, floor, shelving, doorframes, windowsills, doorpushes, splashbacks and sinks) spanning 7 sampling locations including outdoor (shrubs, borders and car park) and indoor (foyer, hallway, kitchen and bathroom) locations (Fig. 1A). Interrogating microbial communities longitudinally facilitated this study to account for changes in temperature, humidity and occupancy (using CO_2_ level as a proxy) throughout the construction process and over seasons. This enabled quantification of the true impact of increasing occupancy within the building after the build was commissioned (Fig. 1B).

### High quality bacterial community sequences can be gained from swabbing surfaces

Sequencing of the V4 region of the 16S rRNA gene yielded a median 1.76 × 10^5^ bacterial reads per sample (IQR: 4.64 × 10^3^–2.58 × 10^5^) and identified 568 different bacterial taxonomic units. Microbial samples had significantly greater library sizes than swab (median = 4.5; IQR = 3.25–5.75; P = 0.01), extraction kit (median = 2.9 × 103; IQR = 1.2 × 103–1.3 × 104; P = 0.003), and sequencing control samples (median = 38; IQR = 37–76: P = 0.0007) (Supplementary Fig. 1). Overall community composition of samples and controls were also significantly different (R^2^ = 0.45, P = 0.001).

### Built environment bacterial communities show seasonality

Temperature, relative humidity and CO_2_ levels were significantly associated with alpha diversity measures of bacterial richness and Shannon diversity in microbial samples (P < 0.05; supplementary Fig. 1). Parabolic relationships were observed between temperature, humidity and alpha diversity. Lowest alpha diversity in microbial communities occurred at temperatures between 10 and 20 °C and relative humidity between 50 and 60%. Additionally, date of sampling was significantly associated with rarefied bacterial richness (P = 0.004) and diversity (P = 0.008).

**Figure 2:**
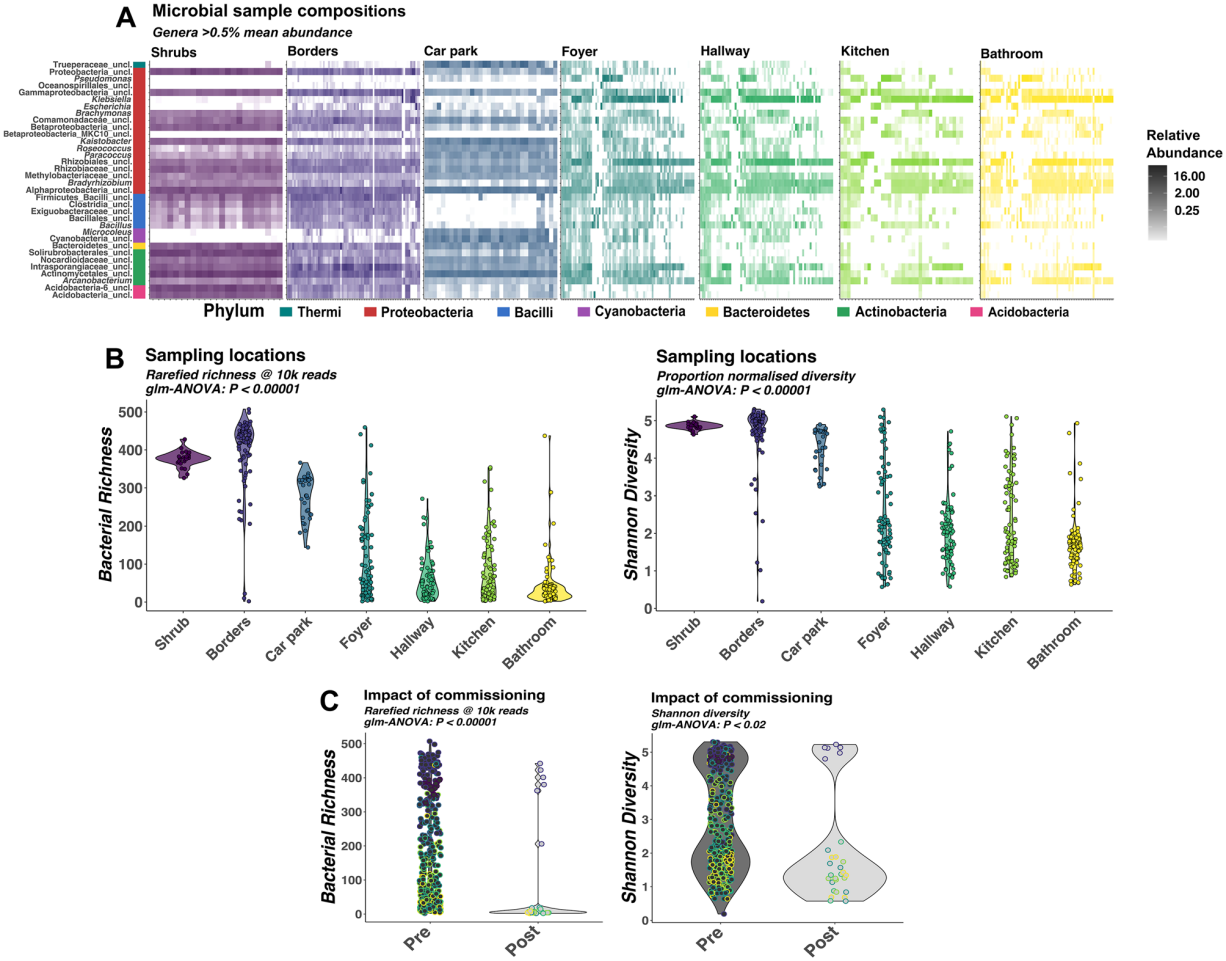
illustrates the overall community compositions of samples analysed in this study. Each tile in the heatmap represents an individual bacterial genus (**A**). Only genera with mean abundance across all samples > 0.5% were included in each panel and arranged along y axis by phylum. Samples were arranged on the x axis according to the sampling timepoint with earliest samples collected on the left and latest on the right within each panel. Intensity of tile colour indicates the relative abundance of the genus in that sample. Sample diversity and richness are illustrated as violin plots across locations (**B**) and timepoints (**C**), where individual samples are represented by points and violin width represents density of samples at that richness/diversity value. Sampling location is illustrated by the hue of points or heatmap tiles with outdoor sampling locations represented by darker purple hues and indoor locations represented by lighter yellow hues, as annotated in the heatmaps.

Taken together, these phenomena could be due to seasonal changes in temperature and humidity, although differences in alpha diversity associated with temperature and humidity changes could be attributed to location. Microbial community compositions were determined from all sampling locations (Fig. 2A). Outdoor samples were taken at lower temperature and were similarly more diverse than indoor samples (Fig. 2B; Table 1). Unsurprisingly, bacterial diversity was highest in temperatures of 30 °C and 80% humidity, which are closest to ideal growth conditions for many host-associated microbes.

**Table 1:** Displays results of generalized mixed linear models produced to test the effect of environmental variables on alpha diversity measures of rarefied bacterial richness and normalized bacterial Shannon diversity of samples included in this study.

Analysis of deviance (type II Wald chi-square test										
Variables	Rarefied richness			Shannon diversity						
	Chisq	df	P value	Chisq	df	P value				
Humidity	12.9295	1	0.0003	20.2943	1	< 0.0001				
Temperature	12.2812	1	0.0005	16.7282	1	< 0.0001				
CO_2_	5.02	1	0.025	20.6419	1	< 0.0001				
Location	268.3375	6	< 0.0001	188.4859	6	< 0.0001				
Object type	266.3059	7	< 0.0001	170.9299	7	< 0.0001				
Commissioning	21.9153	1	< 0.0001	17.8579	1	< 0.0001				
Date	8.1150	1	0.004	6.8884	1	0.008				

Pairwise estimated marginal means (averaged over season and commissioning variables)										
Locations	Rarefied richness					Shannon diversity				
	Estimate	SE	df	T ratio	P value	estimate	SE	df	T ratio	P value
Shrubs—borders	− 0.292	0.65	408	− 0.45	0.9994	2.3965	0.72	408	3.328	0.0165
Shrubs—car park	0.299	0.184	408	1.624	0.6671	0.7394	0.21	408	3.518	0.0087
Shrubs—foyer	1.476	0.661	408	2.232	0.2805	4.0827	0.735	408	5.557	< 0.0001
Shrubs—hallway	2.011	0.653	408	3.08	0.0356	4.3893	0.731	408	6.003	< 0.0001
Shrubs—kitchen	1.726	0.652	408	2.647	0.1149	4.1396	0.728	408	5.689	< 0.0001
Shrubs—bathroom	2.022	0.652	408	3.102	0.0333	4.2858	0.731	408	5.863	< 0.0001
Borders—car park	0.591	0.64	408	0.924	0.9686	− 1.6572	0.705	408	− 2.35	0.2232
Borders—foyer	1.768	0.178	408	9.924	< 0.0001	1.6862	0.183	408	9.228	< 0.0001
Borders—hallway	2.303	0.163	408	14.159	< 0.0001	1.9928	0.172	408	11.561	< 0.0001
Borders—kitchen	2.018	0.152	408	13.262	< 0.0001	1.7431	0.159	408	10.931	< 0.0001
Borders—bathroom	2.314	0.172	408	13.466	< 0.0001	1.8893	0.181	408	10.443	< 0.0001
Car park—foyer	1.177	0.651	408	1.807	0.5443	3.3434	0.72	408	4.642	0.0001
Car park—hallway	1.712	0.643	408	2.662	0.1108	3.6499	0.717	408	5.093	< 0.0001
Car park—kitchen	1.427	0.642	408	2.222	0.2859	3.4003	0.713	408	4.768	0.0001
Car park—bathroom	1.723	0.642	408	2.684	0.105	3.5465	0.716	408	4.95	< 0.0001
Foyer—hallway	0.535	0.115	408	4.634	0.0001	0.3066	0.127	408	2.416	0.1944
Foyer—kitchen	0.25	0.118	408	2.116	0.3449	0.0569	0.131	408	0.435	0.9995
Foyer—bathroom	0.546	0.166	408	3.279	0.0193	0.2031	0.177	408	1.145	0.9136
Hallway—kitchen	− 0.285	0.113	408	− 2.516	0.1561	− 0.2497	0.127	408	− 1.964	0.4391
Hallway—bathroom	0.011	0.153	408	0.072	1	− 0.1035	0.166	408	− 0.623	0.996
Kitchen—bathroom	0.296	0.145	408	2.042	0.3898	0.1462	0.154	408	0.949	0.9641

Generalised mixed linear models were built including humidity, temperature, CO_2_, and date as continuous variables and location, object type and commissioning as categorical variables. Pairwise estimated marginal means were calculated for locations to identify significant differences in alpha diversity between sampling locations and corrected for multiple hypothesis testing with Tukey’s method. (Chisq = Chi-squared statistic; df = degrees of freedom; SE = standard error).

### The impact of occupancy and materials is greater than seasonality on the built environment microbiota

Temperature and humidity changes between seasons may explain some of the longitudinal differences in bacterial richness and diversity observed. However, the impact of commissioning the building on both bacterial richness and diversity (P < 0.001), were greater than those of seasonality. Significantly reduced richness and diversity was observed after the building was commissioned (Fig. 2C).

In addition to seasonal environmental factors and commissioning of the building, sampling locations and object type (P < 0.001) were also significantly associated with rarefied bacterial richness (Fig. 2B). Outdoor locations were significantly richer and more diverse than indoor locations (Table 1). Bacterial communities of shrubs and borders specifically, were significantly more diverse than all indoor locations (P < 0.001).

Several bacterial genera commonly found in soils including *Solirubrobacterales, Kaistobacter* and *Nocardioidaceae* spp. were more abundant in outdoor environments than indoor environments, which harbored more human associated bacteria such as *Klebsiella, Pseudomonas* and *Escherichia* spp. (Fig. 2A).

### Outdoor communities show greater stability over time

**Figure 3:**
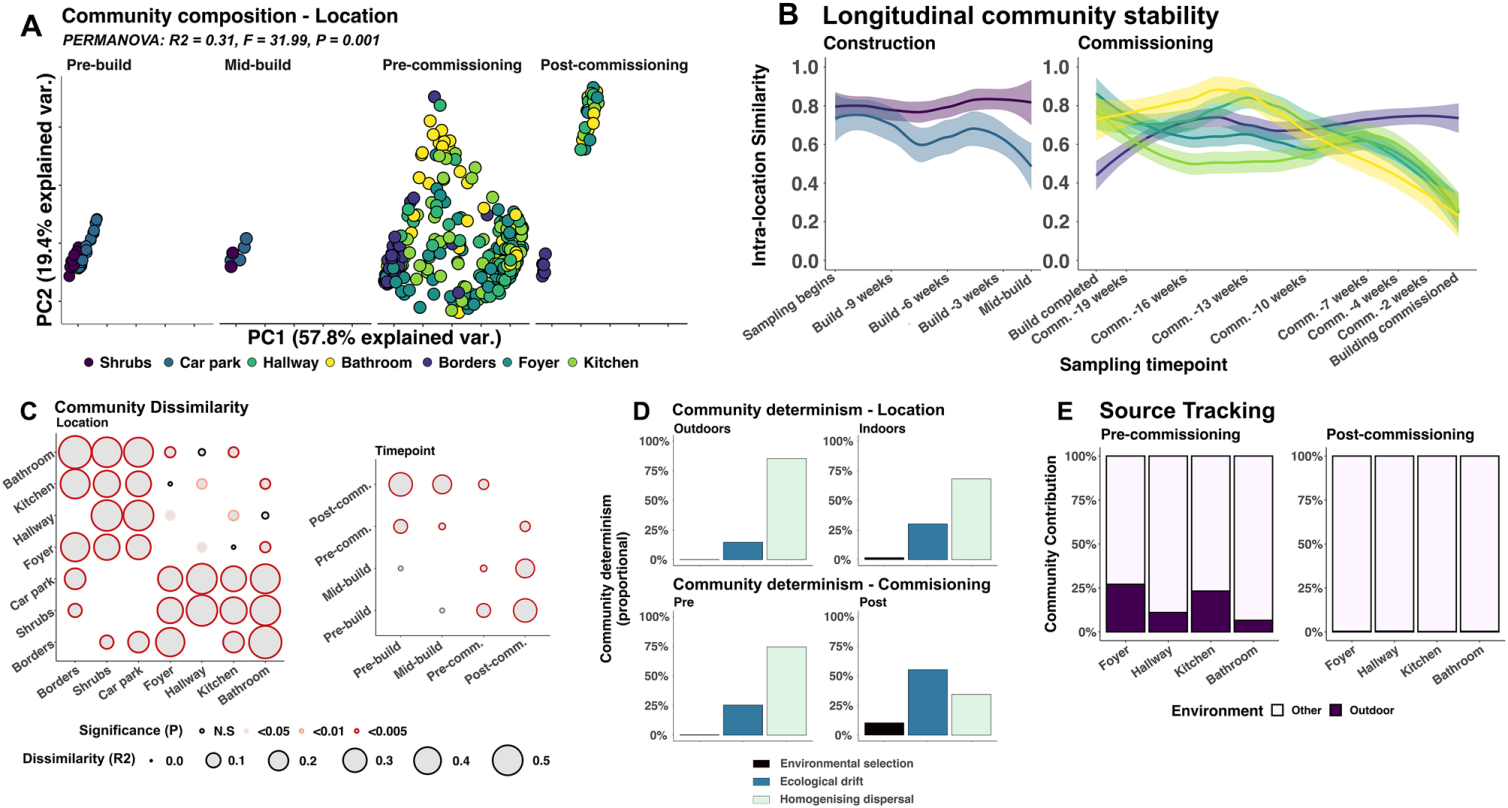
Illustrates overall community structure of samples analysed in this study. Bray–curtis dissimilarity was used to ordinate samples across each of the sampling timepoints (**A**). Each point represents an individual sample and is coloured by location. Longitudinal sample dissimilarity is represented by line graphs (**B**) coloured by the same logic. Lines represent best-fit for inter-sample community similarity at each location with shaded areas representing the 95% confidence interval. Community dissimilarity between communities from all locations and over time are illustrated in bubble plots (**C**). Size of bubbles indicated dissimilarity between two comparators with significance indicated by colour of bubble outline. Raup-Crick similarity between samples was used to define impacts of community determinism on overall community structure (**D**). Bars represent the proportional impact of each phenomenon on samples collected, stratified by locations and timepoints. Influence of outdoor environments on the community structure of indoor communities was identified by SourceTracker (**E**). Sampling timepoint: Pre-comm. = Pre-commissioning; Post-comm. = Post-commisioning.

Compositions of bacterial communities were significantly associated with sampling location (R_2_ = 0.31; F = 31.99; P = 0.001) and timepoint (R_2_ = 0.16; F = 26.70; P = 0.001). The greatest community dissimilarity across locations was observed between outdoor and indoor sampling locations, all of which were significantly dissimilar (R_2_ > 0.32; P < 0.005; Fig. 3A,C).

Indoor bacterial communities shared high similarity immediately following construction (Median = 0.96, IQR = 0.94–0.97; Fig. 3A,B) but became increasingly divergent over time, with many showing very little intra-location similarity following commissioning (Median = 0.05, IQR = 0.01–0.17; Fig. 3A,B). Conversely, outdoor communities showed far more temporal stability. While they were not as similar as all indoor communities immediately following construction, outdoor communities remained consistently similar throughout the sampling period (Median = 0.73, IQR = 0.62–0.81; Fig. 3A,B). The influence of sampling location and commissioning the building are further reflected in the within-group dissimilarity between sampling locations, where indoor bacterial communities were significantly more dissimilar than outdoor locations (P < 0.0001) (Supplementary Table 2).

Community differences between indoor and outdoor surfaces were largely due to the community flux observed in indoor communities over time. As such, indoor communities became increasingly dissimilar to outdoor communities following completion of construction and subsequent commissioning of the building (R_2_ = 0.47; P = 0.001; Fig. 3C). Prior to commissioning, bacterial genera were widely dispersed between outdoor and indoor sampling locations (defined as homogenising dispersal). Homogenising dispersal accounted for 74% of community determinism before commissioning but reduced to 34% after commissioning. By contrast, environmental factors shaping microbial communities (defined as environmental selection) increased from 0.3 to 10.5% following commissioning of the building (Fig. 3D). This was paired with a significant reduction in the mean proportional influence of outdoor microbiota on indoor communities from 17 to 0.004% (P = 2.04 × 10^–15^; Fig. 3E). Increased environmental selection can be attributed to human-associated factors which escalated after commissioning as locations around the building began to be used for their individual, specific purposes.

### Soil-associated metabolic generalist bacteria are replaced by specialists and human associated bacteria as environmental selection increases

**Figure 4:**
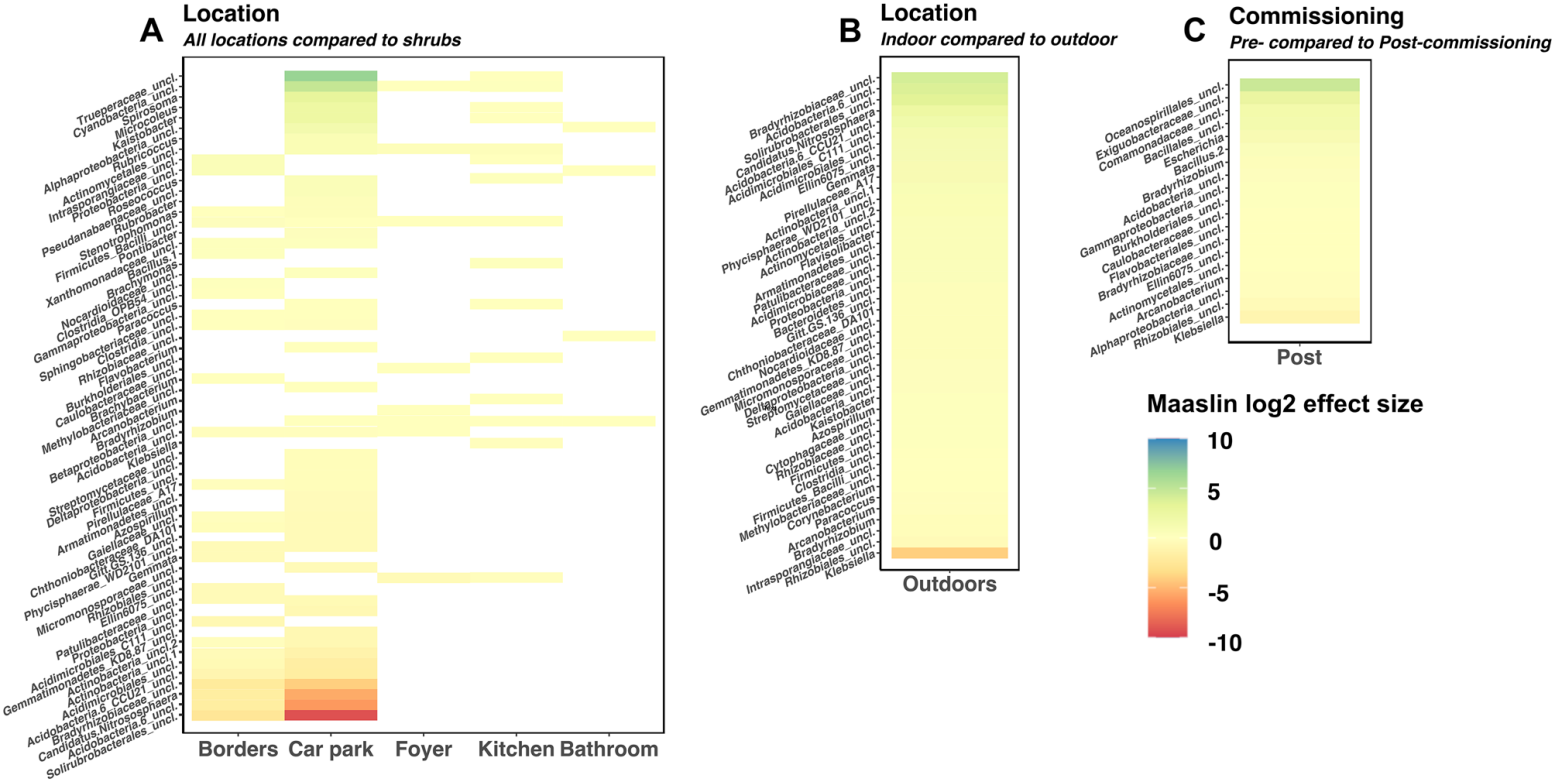
Significant associations of bacterial genera with locations and sampling timepoints. Genera included were significantly associated (Maaslin2 qval < 0.25) with at least one of the environmental variables tested. Only genera with a coefficient of variance > 0.005 are included in each panel. Tile colour indicates the Maaslin2 effect size of each environmental variable on genus proportional abundance (reduced = red; increased = blue). Sampling locations were included as fixed effects with ‘Shrubs’ as the comparator variable (**A**). Indoor communities were compared to outdoor communities (**B**) and pre-commissioning communities were compared to post-commissioning communities (**C**).

Increased environmental selection brings external factors which shape bacterial communities. Environmental selection increased after construction was completed and the building was commissioned in this study. Increased footfall in indoor environments along with introduction of specific cleaning and usage regimes had measurable effects on the taxonomic compositions observed across sampling locations (Fig. 4A,B). *Klebsiella *spp. are common, human-associated, enteric commensals that were significantly associated with indoor environments and pre-commissioning timepoints (Fig. 4B,C). Soil-associated Actinobacteria such as *Solirubrobacterales* and *Gaiellaceaea *spp. as well as the nitrogen fixing *Bradyrhizobiaceae* and the archaea *Nitrososphaeraceae *spp. were all significantly associated with outdoor environments while halotolerant *Oceanospirillales *spp. were significantly associated with pre-commissioning communities.

## Discussion

This study represents the first to our knowledge to characterize the original acquisition and subsequent factors influencing community structure of any built environment microbiota to such depth. The study deliberately included a longitudinal sampling aspect to account for seasonality of microbial communities which we found to be significantly linked with temperature, humidity and CO_2_ concentrations. Furthermore, by collecting microbial samples within the built environment site prior to construction starting, we were able to benchmark any subsequent microbial communities with that of the indigenous or well-established microbes that preceded them. The importance of doing so became increasingly apparent upon analysing the data, where we highlight the relative stability of outdoor, pre-established, microbial communities by comparison to the highly turbulent indoor communities developing following completion of the structure.

A previous study highlighted the importance of space utilization on bacterial community composition^5^. Surprisingly, Chase et al*.,* found surface material has minimal influence on microbial community composition^25^, which is consistent with the findings of this study. We found microbial communities across multiple surfaces in different indoor locations to cluster according to the room they were in and the usage patterns, rather than according to material surface. These results may be a product of the insensitivity to microbial viability of the sampling methods used to assay these environments^27,28^ as other studies have found surface material to be important in shaping built environment microbial communities^29^. To facilitate high-throughput processing of the microbiomes interrogated here no viability determinism was performed, therefore the presence of relic DNA from non-viable bacteria cannot be discounted in this, or previous, studies. Approaches that can differentiate viable from non-viable microbes such as culturing^30,31^ or use of viability dyes^32^ may serve to answer these questions in the future.

Roughly three months after construction of the built environment investigated in this study, a regular cleaning regime was established. The introduction of biocidal agents at this time matched well with the timepoints at which indoor microbial communities begin to specialise, converging at the commissioning date in communities displaying very high degrees of environmental selection. This is not the first time this phenomenon has been observed. Flores et al*.* showed specific communities of bio-film forming Gram-negatives associated with regularly cleaned areas^6^, while others have shown homes in more urban areas, which are cleaned more frequently, have reduced diversity^12,33^. We found increased abundances of *Escherichia,* and multiple Bacilli following commissioning and establishment of cleaning regimes within the built environment we explored here. Multiple antimicrobial resistant strains of *Escherichia* are reported in clinical studies while Bacilli are known to produce glycosphingolipids^34^ which stabilise the bacterial membrane and confer resistance to polymixin class antibiotics^35^. These data add to the growing evidence base that where communities are regularly exposed to biocidal interventions, the intended sterility may not be achieved. Instead, frequent cleaning may simply act as a selective pressure for microbes resistant to the killing mechanisms employed^36^, resulting in more robust survivor communities.

This study found indoor microbiota to become increasingly dissimilar to the outdoor communities that seeded them over time. A longitudinal study of the home microbiota of 7 families over 6 weeks found bacteria in indoor spaces to be predominantly of human origin^37^. The same study also found that the human associated microbial signatures in indoor environments diminished as quickly as three days after a human vacated their home. In the study by Lax and colleagues, however, the influence of human microbes had already been established in each home. Our study identified that indoor microbiota were still developing as late as 34 weeks after completion of the construction of the building. This phenomenon may be specific to commercial properties and related to a lag period between completion of construction and commissioning of the building. As such, these findings may not be consistent with those observed in residential properties, which are generally occupied sooner following completion. Likewise, in family homes the influence of one individual on microbial community structures may be greater due to them spending substantially more time in their own home compared to visitors to the completed structure investigated here. Nonetheless, as in the study by Lax et al. we found the indoor microbiota to be significantly enriched with multiple commonly human-associated microbes including *Klebsiella* and *Escherichia *spp. after commissioning^37^.

Covid-19 lockdowns impacted our ability to consistently collect longitudinal microbial samples and record environmental variables throughout this study. As a result, numbers of samples and observations available at each timepoint are unbalanced. While the statistical methods employed were specifically chosen to address these limitations, by comparing ranked means rather than actual values, it must be noted that the strength of conclusions drawn on temporal microbiota development could be strengthened via a balanced study design. It can be difficult to identify contaminant bacterial features in low-biomass environmental samples during targeted amplicon sequencing, especially since many common contaminants observed in microbial community analyses originate from soils and watercourses^38,39^, both of which are sampled locations in this study. To address this we have sequenced blank swabs, kit negative and sequencing reagent controls as recommended by previous studies assessing the influence of contamination. Sample library size and compositions were significantly different to controls, demonstrating the legitimacy of bacterial features identified in this study.

Through high-throughput, longitudinal sampling of a single, newly-built structure, this study demonstrates the impact of human interaction and interventions on the microbiota of the built environment. A transient community of bacteria, initially dominated by those from outdoor environments and showing high degrees of homogenizing dispersal, precedes more location specific communities once humans begin to inhabit and interact with spaces in the built environment. These microbial community changes are likely due to increasing environmental selection.

## Methods

### Bacterial sample collection

The OME is an experimental building constructed and commissioned as part of the Hub for Biotechnology in the Built Environment. Throughout the construction process between December 2019 and November 2021, and until the official launch in June 2022, a total of 439 microbial samples were collected from multiple surfaces on the OME site. The sites included both indoor and outdoor surfaces, consisting of natural and synthetic materials orientated both vertically and horizontally.

The longitudinal nature of this study enabled sampling of *The OME* site before ground was broken during the construction process. At this point, only outdoor samples were taken, as no building existed to take samples inside. Supplementary Table 1 describes the location, material and construction timepoints spanned during sampling at each site.

Samples were collected by swabbing a 10 cm^2^ area for 30 s using Microgen Path-check swabs (Novacyt, UK). Immediately following each collection, batches of swabs were transported back to laboratories at Northumbria University and processed for bacterial nucleic acid isolation. At each sampling timepoint temperature, humidity, and CO_2_ levels were recorded using an ELKLIV SR-SIO Indoor CO_2_ meter. Recordings were taken every 90 s for a minimum of 15 min across outdoor, foyer, kitchen and hallway locations. Readings from the ELKLIV SR-SIO meter were validated by cross-referencing against those from a ParticlesPlus Model 7501 Remote Particle counter.

### Nucleic acid isolation, library preparation and sequencing

Swab tips were cut off using sterile scissors and suspended in 1 × PBS (1 ml) in sterile microfuge tubes (2 ml). Tubes were vortexed horizontally at high speed for 25 min to release biological material before swab tips were discarded and remaining solution was centrifuged (5000 xG, 30 min) to pellet bacterial cells. The supernatant at this stage was discarded and bacterial pellets were resuspended in DNeasy bead solution (750 µL, (QIAGEN, DE)). Nucleic acids were isolated from resulting bacterial suspension using DNeasy PowerLyzer PowerSoil Kit (QIAGEN, DE) according to manufacturer’s instruction save for an extended bead beating time of 25 min to ensure full lysis of gram positive bacteria and 15 min incubation of filters at room temperature after addition of elution buffer to increase nucleic acid yields. An isolation kit negative control (1 × PBS, 1 ml) was processed with each batch of swab samples.

Sequencing was performed by NUOMICS DNA Sequencing Facility (Northumbria University, Newcastle, UK). Bacterial communities were determined by targeted sequencing of the V4 region of the 16S rRNA gene using primers 515F and 806R^40^. Prepared genomic libraries were sequenced on the Illumina MiSeq using V2 2 × 250 bp chemistry (Illumina, UK). A sequencing negative control (nuclease free water) was processed with each plate of samples during library preparations.

Raw sequencing output consisted of fastq files which were filtered to include only those with quality phred scores of > Q30. Taxonomic classification of resulting fastq files was performed in QIIME2^41^. Briefly, paired end reads were merged^42^, trimmed to a maximum 253 bp^43^ and clustered at 97% similarity^42^. Chimeric reads were screened and removed^44^ before taxonomy was assigned using custom-trained classifiers, aligned to the Greengenes2 database (v2022.3)^45^. Features classified at sub-phylum level taxonomy were culled and taxonomic features were merged at the genus level before biom files were exported^46^ and compositional analyses were performed in R^47^ using the phyloseq package^48^.

### Statistical analyses

Comparisons between means of continuous data were performed by Kruksal-Wallis (KW) rank sum test. Bacterial feature abundances were normalized by converting raw counts to proportional abundances per sample. Alpha and beta diversity were calculated using vegan^49^. Alpha diversity metrics were calculated from raw feature counts as rarefied (10 k reads) bacterial richness and from proportionally normalized abundances as Shannon diversity. Beta diversity was calculated from proportionally normalized abundances as Bray–Curtis dissimilarity and from raw feature counts as rarefied (10 k reads) Raup-Crick similarity between samples.

Generalised linear mixed models (glm) were built to determine associations of environmental factors including (as fixed effects): temperature, humidity, CO_2_ levels, building location, object type, commissioning and sampling date on alpha diversity^50^. Least-square means of significantly associated environmental factors were compared^51^ with multiple pairwise comparisons and corrected where appropriate using Tukey’s method.

PERMANOVA was performed to identify significant associations between Bray–Curtis dissimilarity and environmental factors including location and sampling timepoint. Where significant associations were observed, pairwise PERMANOVA^52^ was used to identify classes of each variable with significantly different community composition. Comparisons were corrected for multiple hypothesis testing using Bonferroni’s method. Within group dispersal for sampling locations and timepoints was assessed using PERMDISP. ANOVA was used to compare mean distances from individual sample compositions to group Euclidean centroids and Tukey’s HSD identified significant differences in dispersal between location/timepoint groups.

Community determinism was compared by rescaling Raup-Crick similarity to values between − 1 and + 1. Environmental selection, where environmental factors have large determinative effects on the community composition of samples, was defined as samples with Raup-Crick similarity values < − 0.95. Alternatively, homogenising dispersal, describes situations where environmental pressures have a minimal impact on community composition, with multitudes of shared features between samples. This was defined as samples with Raup-Crick similarity values > 0.95. Raup-Crick similarity values between − 0.95 and 0.95 were defined as ecological drift, being the case where environmental factors influenced microbial populations but did not prevent transfer of bacteria between them.

The influence of outdoor on indoor communities was determined by SourceTracker^53^ with outdoor locations defined as “sources” and indoor locations as “sinks”. Differential bacterial features between sampling locations and timepoints were identified using Maaslin2^54^. Results were visualized with ggplot2^55^.

### Supplementary Information

Supplementary Figures.

Supplementary Table 1.

Supplementary Table 2.

## Data Availability

Data are available on the European Nucleotide Archive under accession PRJEB58762.
