# Supplementary material for: Built environment microbiomes transition from outdoor to human-associated communities after construction and commissioning
Source: Sci Rep. 2023 Sep 22;13:15854. doi: 10.1038/s41598-023-42427-0 (PMC10516947; doi:10.1038/s41598-023-42427-0)
Supplement: Supplementary file 1 — Supplementary Figures. [file 41598_2023_42427_MOESM1_ESM.docx]

Supplementary figures

Built environment microbiomes transition from outdoor to human-associated communities after construction and commissioning.

Gregory R Young, Angela Sherry, Darren L Smith

Supplementary figure 1


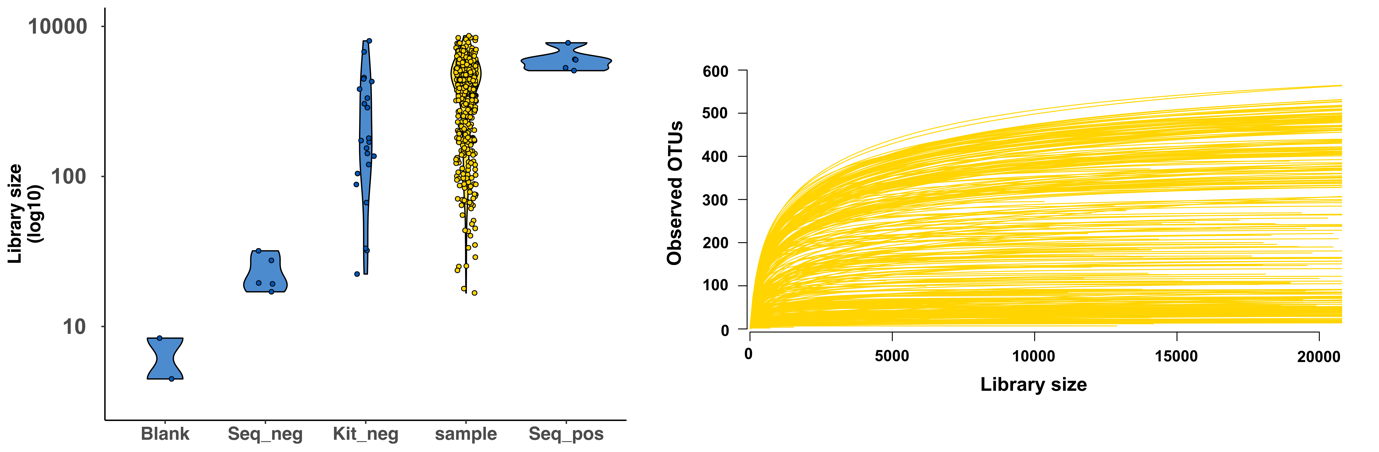


*Supplementary figure 1 illustrates library sizes yielded by 16S rRNA gene sequencing. Each point in the left panel represents an individual sample and density clouds illustrate the distribution of library sizes within each group.*

*Environmental samples yielded significantly greater library sizes (median = 1.8x10^4^; IQR = 4.6 x10^3^ – 2.9 x10^4^) than blank (median = 4.5; IQR = 3.25 – 5.75; P = 0.01), sequencing negative (median = 38; IQR = 37 – 76: P = 0.0007), and kit negative controls (median = 2.9 x10^3^; IQR = 1.2 x10^3^ – 1.3 x10^4^; P = 0.003).*

*Rarefaction of samples (right panel) shows sufficient sampling depth was achieved at 10k reads to characterize the full taxonomic richness of any given sample.*

Supplementary figure 2

*
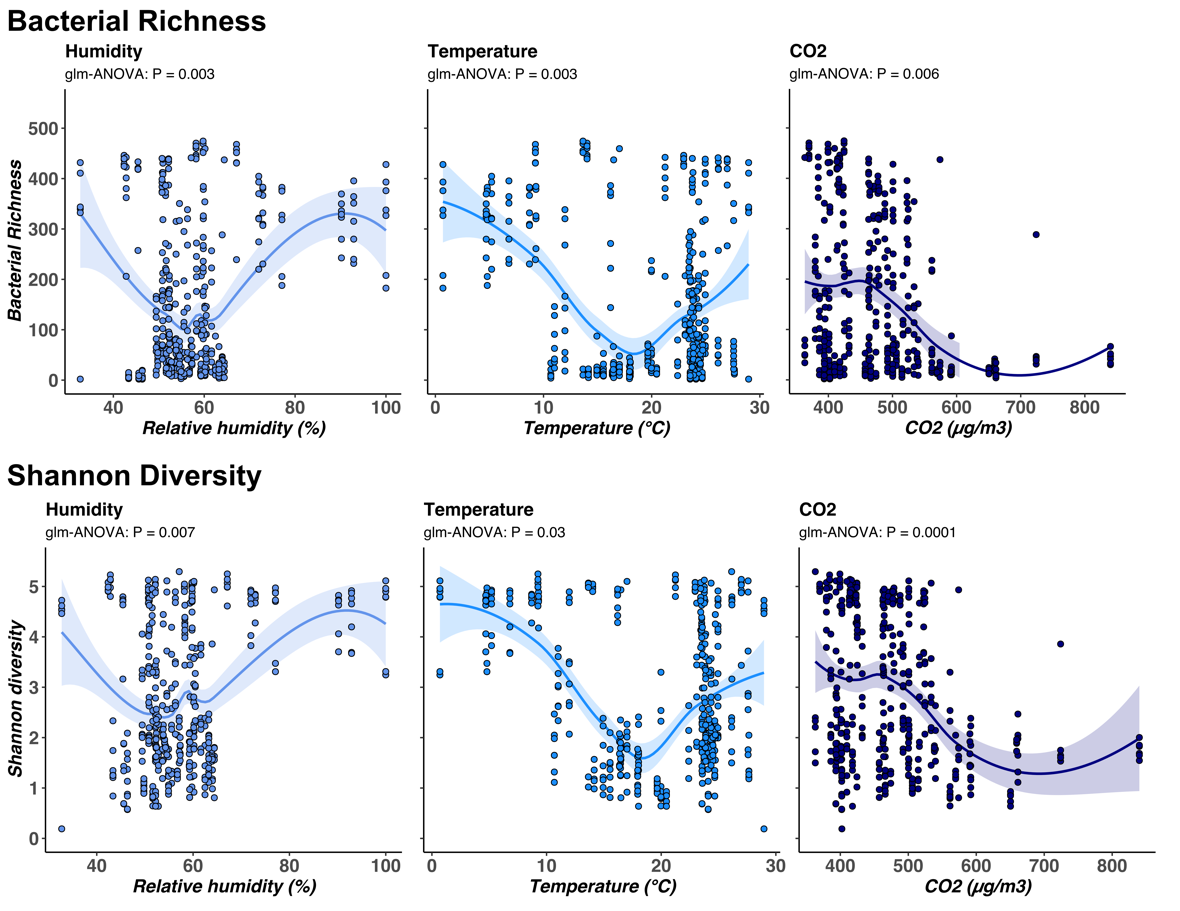
*

*Supplementary figure 2 illustrates associations between relative humidity, temperature and CO_2_ levels with alpha diversity of microbial communities. Alpha diversity measures were calculated as bacterial richness (rarefied at 10k reads) and Shannon diversity (proportionally normalised). Each point represents an individual sample with environmental parameters plotted on the x axis and alpha diversity measures plotted on the y axis. Lines on each panel represent the best-fit for each relationship with shaded areas extending to the 95% confidence intervals.*
